# Mobile Typing as a Window into Sensorimotor and Cognitive Function

**DOI:** 10.3390/brainsci15101084

**Published:** 2025-10-07

**Authors:** Lorenzo Viviani, Alba Liso, Laila Craighero

**Affiliations:** 1 Department of Medical Sciences, University of Ferrara, via Fossato di Mortara 64/B, 44121 Ferrara, Italy; lorenzo.viviani@unife.it; 2 Department of Neuroscience and Rehabilitation, University of Ferrara, via Fossato di Mortara 19, 44121 Ferrara, Italy; alba.liso@unife.it

**Keywords:** mobile typing, sensorimotor adaptation, skill acquisition, cognitive function, behavioral biomarkers, cortical plasticity

## Abstract

The rapid evolution of human–technology interaction necessitates continuous sensorimotor adaptation to new digital interfaces and tasks. Mobile typing, defined as text entry on smartphone touchscreens, offers a compelling example of this process, requiring users to adapt fine motor control and coordination to a constrained virtual environment. Aligned with the embodied cognition framework, understanding these digital sensorimotor experiences is crucial. A key theoretical question is whether these skills primarily involve adaptation of existing motor patterns or necessitate de novo learning, a distinction particularly relevant across generations with differing early sensorimotor experiences. This narrative review synthesizes current understanding of the sensorimotor aspects of smartphone engagement and typing skill evaluation methods. It examines touchscreen competence, skill acquisition, diverse strategies employed, and the influence of interface constraints on motor performance, while also detailing various sophisticated performance metrics and analyzing different data collection methodologies. Research highlights that analyzing typing behaviors and their underlying neural correlates increasingly serves as a potential source of behavioral biomarkers. However, while notable progress has been made, the field is still developing, requiring stronger methodological foundations and crucial standardization of metrics and protocols to fully capture and understand the dynamic sensorimotor processes involved in digital interactions. Nevertheless, mobile typing emerges as a compelling model for advancing our understanding of human sensorimotor learning and cognitive function, offering a rich, ecologically valid platform for investigating human-world interaction.

## 1. Introduction

The rapid evolution of human–technology interaction has profoundly reshaped how individuals access information, communicate, and work. A clear example of this transformation is the act of writing. Historically rooted in manual penmanship, writing evolved with the introduction of the typewriter, becoming an input method mediated by a mechanical interface. This shift to typewriting necessitated the adaptation of cognitive-sensorimotor functions, including spatial memory for key placement and bimanual coordination [1], thus establishing typewriting as a new functional skill.

The advent of personal computers and the internet dramatically accelerated human-technology interaction, establishing the digital space as a prevalent operational environment. This supported a wide array of activities such as information retrieval, content creation and sharing, email communication, online learning, and job management [2]. In this text-based digital realm, typing solidified as a fundamental skill, enabling efficient engagement with diverse digital services.

The proliferation of smartphones marked further significant development in human-technology interaction. Smartphones, being uniquely portable, lightweight, multipurpose, and constantly connected, have become indispensable tools for managing personal, social, and professional life. Their prevalence is striking; as of early 2024, 5.61 billion people globally use a mobile phone, representing nearly 70 percent of the total world population [3]. The overall number of mobile connections is even higher (reaching 16.73 billion), yet this user figure alone highlights the near ubiquity of these devices. Mobile phones are now the primary means of accessing the Internet for 92.3% users in 2024, with the average user spending 5 h and 23 min on their device daily [3]. This profound integration into daily life and constant engagement through a touchscreen interface has generated significant scientific interest, particularly regarding their potential cognitive and psychological effects, including impacts on attention, memory, self-regulation, and addiction [4,5].

While cognitive and psychological impacts of technology use have received substantial attention, the influence of touchscreen interfaces on the sensorimotor system remains an understudied area. Mobile typing, specifically defined as text entry on smartphone touchscreens, is a sensorimotor activity that is highly shared, frequent, and ecologically relevant. Indeed, evidence from other research domains strongly suggests that repetitive motor experiences—like those inherent in mobile typing—can significantly reshape cortical organization. In fact, extensive sensorimotor engagement is known to result in neural modifications within cortical areas associated with fine motor control [6,7,8,9]. This perspective aligns with the embodied cognition framework, which posits that cognitive processes are deeply rooted in the interactions of the body with its environment [10,11,12]. Applying this perspective to the digital age, it is plausible that the extensive and often qualitatively different sensorimotor experiences of interacting with digital devices have reshaped human cognition and its neural substrate. This is particularly relevant given that, unlike traditional sensorimotor experiences which remained relatively stable across generations (e.g., to grasp a cup), the rapid evolution and widespread adoption of digital technology have introduced entirely new patterns of interaction. This has led to a critical generational distinction: older individuals have primarily established their sensorimotor repertoires through interaction with the physical world, while younger generations have formed theirs by acting concurrently in both physical and digital environments. Such a fundamental divergence could influence whether mobile typing skills primarily involve adaptation and generalization of existing motor patterns or if they necessitate significant de novo learning, potentially leading to qualitatively distinct motor control strategies, cognition, and neural substrates across age groups.

Until recently, studies of the sensorimotor system predominantly required participants to execute goal-directed actions such as grasping an object for use or for transfer [13,14], or to observe such actions being performed [15]. Techniques like response time measurement, 3D motion capture and analysis, brain imaging, transcranial magnetic stimulation, and electrophysiological methods have been widely used to investigate these competencies. These studies have yielded a relatively clear theoretical framework for how actions are planned and executed based on their ultimate intention or purpose [15,16,17]. However, such controlled laboratory studies are challenging to implement in truly ecological situations (e.g., during daily life) or to repeat longitudinally within the same individual to examine modifications due to aging or disease progression. In contrast, mobile typing—given its high frequency, sharing across generations, and ecological nature—offers a unique opportunity as a model for studying motor skill acquisition and development. Its analysis could be conducted across diverse populations (e.g., varying ages, such as Digital Natives vs. Digital Immigrants as discussed by Pensky [18]), in different contexts (e.g., content consumption vs. communication, reflecting distinct digital intentions [19]), and under varied motivations (e.g., prompted vs. spontaneous typing).

However, despite its potential, the sensorimotor characteristics of mobile typing remain poorly understood. In particular, there is a notable lack of research into its subtle kinematic features, e.g., peak velocity, acceleration, deceleration, and their relative timings of single typing action. A single typing action is hereby defined as the goal-directed movement of a finger from an initial key position (e.g., a letter key or the spacebar) to depress the intended target key. These kinematic parameters measured during interaction with physical objects (e.g., grasping a cup, the reaching movement from a starting position until the object is held), are highly sensitive to changes influenced by context, age, or purpose [14,15], and could provide significant insights for building accurate and predictive models of action planning and execution specific to mobile typing. To effectively pursue such studies, it is essential to first understand the current state of the art and identify which variables are known to interfere with task execution (e.g., typing modality, presence of textual suggestions, the specific purpose of the typing task, etc.).

The aim of the present narrative review is, therefore, to provide a synthesis of the relevant literature on smartphone text entry, critically assessing the current state of knowledge across its diverse components and the methods used to study them, and to identify crucial knowledge gaps that warrant further investigation. This review specifically synthesizes current understanding regarding the unique sensorimotor and cognitive aspects of mobile typing, alongside the diverse methodologies employed to study it, and highlights its emerging role as a behavioral biomarker. By consolidating this scattered literature into a comprehensive knowledge framework, our primary goal is to aid in planning future experiments that leverage mobile typing as a compelling model for investigating sensorimotor learning and development, and to guide its utilization as a robust tool for monitoring digital competence, understanding the process of specific skill acquisition across diverse populations and contexts, and detecting any eventual loss of efficiency or early signs of impairments.

## 2. Mobile Typing Skill Acquisition and Development

### 2.1. The Origins of Touchscreen Competence in Infants and Influence on Fine Motor Skills

Fine motor skills (FMS) refer to the coordinated movements of the small muscles in the hands and fingers, often in conjunction with the eyes. In the context of infant and child development, the development of fine motor skills is a crucial milestone, reflecting the maturation of the nervous system, muscle strength, and coordination. These skills are essential for interacting with our environment and typically rely on the ability to perceive objects through touch and vision, as well as proprioception (the sense of position of fingers, hand, and lower arm). This also involves integrating information across these different sensory modalities, known as visual–haptic integration. Within this context, touchscreens present a unique perceptual challenge, acting as flat, two-dimensional (2D) surfaces that offer more interactive possibilities than static photographs, yet less manual exploration than three-dimensional (3D) objects, as highlighted by Ziemer et al. [20].

Research into the development of touchscreen competency in children has been advanced by studies such as Hourcade et al. [21], who analyzed YouTube videos to observe how infants and toddlers use tablets. Their findings revealed a developmental progression in touchscreen interaction abilities related to age. Infants under 12 months old, did not demonstrate a marked ability to utilize apps on tablets, primarily interacting by hitting the screen with a full hand and multiple fingers, and were significantly more likely to use both hands rather than a single hand. Between 12 and 17 months, children began to exhibit moderate ability, starting to interact with a single finger, mainly through tapping. This moderate ability continued into the 18-to-23-month age range, with interactions still centered on single-finger taps, but with a notable increase in drag interactions, and a growing preference for using only one hand. By 24 to 29 months, children demonstrated a moderate ability to both control and understand the apps they were using, with single-finger taps remaining the dominant interaction, but further increases in drag interactions and a more established pattern of single-handed use.

Building upon this understanding of the developmental trajectory of touchscreen interaction, Ziemer et al. [20] experimentally examined how infants explore these interactive surfaces. Their study investigated the manual behaviors of 7–10-month-old and 15–18-month-old infants when presented with touchscreens, photographs, and objects for 30 s each. They categorized manual behaviors into 2D actions (pats and rubs) considered suitable for photographs, 3D actions (grasps and scratches) appropriate for objects, and touchscreen-specific actions (swipes, taps, pinches, and spreads). Their findings indicated a developmental progression in touchscreen competence: while 7–10-month-olds did not differentiate their manual behaviors across the different types of stimuli, 15–18-month-olds exhibited discriminatory behaviors towards screen images, indicating the development of touchscreen competence by at least 15 months.

Research on the broader relationship between touchscreen use and FMS in young children presents a mixed picture. Some studies, using parent reports on a single FMS task (stacking blocks) and retrospective designs, suggest a potential positive association [22] found that earlier touchscreen scrolling was linked to earlier fine motor achievement in infants. Similarly, Souto et al. [23] observed a slight advantage in fine motor skills for young children with tablet-use experience. Moon et al. [24] also reported a positive correlation between smart device usage and fine motor development in three-year-olds. However, other longitudinal research with more comprehensive FMS batteries indicates a potential negative association or differential effects: Martzog & Suggate [25] found that newer media were associated with lesser FMS development over time and proposed that screen-media may lead to a disadvantage in FMS and haptic skills, even while potentially enhancing visual-shape discrimination. Furthermore, two studies from the same research group have highlighted significant negative impacts of regular tablet use on the development of FMS among preschool children [26,27]. One study [26] found that children with frequent tablet use (defined as using a tablet more than once a week for gaming or education) performed significantly worse than non-users on multiple visual perception measures, including visual discrimination, visual memory, spatial relationships, form constancy, and visual figure-ground perception. This study also revealed that frequent tablet users scored significantly lower than non-users in fine motor precision, fine motor integration, and manual dexterity. Related work by the same authors [27] investigated the effectiveness of a 24-week daily tablet-based FMS training program. This tablet program proved less effective than a traditional hands-on manual program and, remarkably, led to a decline in children’s FMS performance when comparing post-test to pre-test scores.

Taken together, these contrasting findings highlight the need to consider methodological differences, the specific aspects of FMS assessed, and the nuances of media usage (e.g., quality of engagement, contextual embeddedness) when interpreting the relationship between touchscreen exposure and fine motor development. The ongoing debate underscores that benefits or detriments may depend more on how devices are used rather than simply how often.

### 2.2. General Typing Skill Acquisition and Its Adaptation to Mobile Devices

Mastering typing, like any learning process, involves a transition from a slow, cognitively demanding approach to the automatization of movements through repeated practice. Sono & Hasegawa [28] identified three crucial aspects to improve typing ability on physical keyboards: (a) remembering key placement, (b) proper fingering and (c) touch typing. Memorizing key placement allows typists to avoid visual search, thereby increasing typing speed. Utilizing the appropriate finger for each key, which can enhance typing speed and accuracy, reflects the development of internal maps linking the keyboard layout to the finger movement coordination. Ultimately, mastering these two aspects leads to a touch-typing style where visual monitoring of the keyboard is no longer necessary.

**Figure 1:**
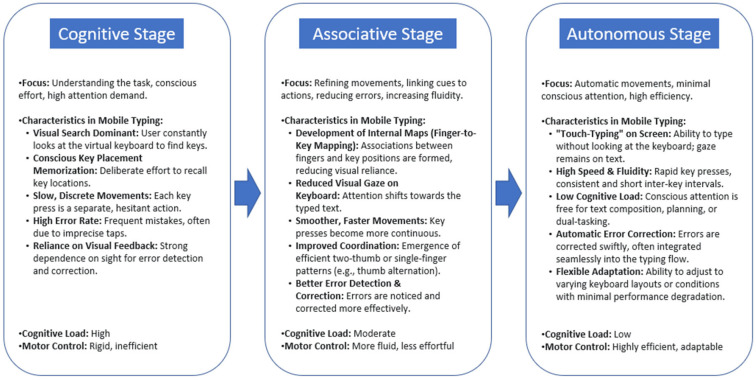
A sequential diagram illustrating the three stages of motor skill acquisition—Cognitive, Associative, and Autonomous—as adapted from Fitts & Posner’s model [29]. Each stage is characterized by distinct cognitive and motor demands, with specific manifestations in the context of mobile typing on touchscreen devices. This progression highlights the shift from conscious, effortful movements to automatic, efficient performance, emphasizing the underlying processes of learning and adaptation.

This proposal aligns with the Fitts & Posner [29] classic skill acquisition model, who characterized performance by three sequential stages: cognitive, associative, and autonomous. Figure 1 illustrates how these stages manifest specifically in the context of mobile typing. In the Cognitive Stage, learners focus on understanding the task, relying heavily on visual cues. For mobile typing, this involves active visual search for keys and conscious memorization of the keyboard layout, aligning with Sono & Hasegawa’s [28] ‘remembering key placement’. Movements are typically slow, discrete, and error-prone, requiring high cognitive demand. As users progress to the Associative Stage, movements become more refined and efficient. This stage sees the development of internal ‘finger-to-key’ mappings, allowing for more ‘proper fingering’ [28] and reduced dependence on constant visual monitoring of the keyboard. Typing becomes smoother and faster, with a decrease in error rates. Finally, in the Autonomous Stage, typing becomes largely automatic and highly efficient, characterized by what can be termed ‘touch-typing’ on the screen. Here, visual attention is freed from the keyboard, movements are fluid and rapid, and cognitive load is minimal, allowing for effortless text composition. This progression underscores the fundamental shift from effortful, visually guided actions to automated, precise motor patterns, which is critical for achieving proficiency in mobile text entry.

Traditional understandings of typing expertise have largely been shaped by studies focusing on trained typists employing ten-finger touch typing systems [30,31,32,33,34,35]. However, recent studies challenged this perspective by examining the everyday typing techniques and behaviors of self-taught individuals [36,37]. In a transcription task comparing self-taught and formally trained typists, Feit et al. [37] found no significant difference in typing speed. Utilizing motion capture technology to measure performance, gaze behavior, and movement strategies, their analysis identified three key predictors of high typing performance, irrespective of formal training: consistent finger-to-key mapping, proactive preparation for upcoming keystrokes, and minimizing global hand motion. This finding challenges the long-held belief that formal training is indispensable for achieving speed typing proficiency, suggesting new directions for optimizing keyboard layouts and adaptive input methods. Examining deeper into typing strategies, Feit et al. [37] study revealed diverse motor approaches between trained and self-taught typists. Notably, self-taught typists demonstrated a greater reliance on visual feedback, dedicating more time to directly observing the keyboard. Further analysis through hierarchical clustering of individual finger-to-key mappings uncovered distinct typing styles. While some self-taught individuals adopted strategies resembling trained touch typing, others utilized less conventional yet effective methods, highlighting the remarkable adaptability of users in developing personalized typing techniques.

Further exploring the underlying motor mechanisms of typing, Cerni et al. [38] investigated whether manual coordination patterns observed in touch-typing on standard keyboards are also present in mobile typing. In their study, skilled touch-typists were divided into two groups based on their mobile typing habits: a “two-hands” group using thumbs and a “one-hand” group using a single index finger. Participants performed typing-to-dictation tasks on both standard and mobile keyboards. Remarkably, the study found that the “two-hands” mobile typists exhibited a similar “bimanual advantage” as seen in touch-typing—manual alternation (using different thumbs for successive keystrokes) was faster than manual repetition (using the same thumb). This advantage, characterized by decreased inter-keystroke intervals with increased bimanual transitions, was observed in both standard and mobile keyboard tasks for the “two-hands” group. Conversely, the “one-hand” mobile typists showed an opposite pattern in mobile typing, lacking the bimanual advantage. Furthermore, the study found that other effects known to influence motor aspects of typing (i.e., bigram frequency effects) were also present in mobile typing for the “two-hands” group, suggesting similar underlying motor processes across keyboard types. These findings indicate that fundamental principles of manual coordination in typing, such as the bimanual advantage and sensitivity to bigram frequency, can generalize to mobile typing when using two thumbs, despite the different physical interfaces. However, when mobile typing is constrained to a single finger, the coordination patterns shift, highlighting the adaptability of typing strategies to interface constraints.

Specifically concerning mobile keyboards, Jiang et al. [39] investigated the learning process tracking the evolution of eye and finger movement strategies as participants typed on a smartphone using QWERTY, statically randomized (SR), and dynamically randomized (DR) keyboard layouts. The researchers demonstrated that mobile typing skill development involves not only motor learning but crucially, the adaptation of visual attention and eye-hand coordination. They observed a significant shift in strategies as users gained familiarity with the layout. Novices in the DR condition heavily relied on visual search, while increased familiarity (SR condition) led to the development of location memory for the keyboard, shaping visuomotor control strategies and influencing speed-accuracy trade-offs and gaze deployment between the keyboard and text entry areas. Jiang et al. [40] also showed that eye-hand coordination strategies differ depending on the typing method. Specifically, two-thumb typists relied less on visual guidance and shifted their gaze between keyboard and text area less frequently than one-finger typists.

Expanding the scope beyond novice-expert dichotomies, Pinet et al. [41] aimed to characterize typing expertise within a large student population. Their large-scale online study involving over 1300 university students investigated factors influencing typing performance, including practice habits and cognitive variables. Their findings suggest that contemporary typing expertise in young adults is better understood as a continuous spectrum, emerging from frequent keyboard use rather than solely from deliberate practice. While deliberate practice did not significantly predict proficiency in this population, key factors differentiating typing skill levels included the amount of daily typing, years of practice, finger usage, and reduced visual attention to the keyboard.

## 3. Mobile Typing Modalities and Interfaces

### 3.1. The QWERTY Keyboard: A Legacy in Mobile Interaction

The QWERTY keyboard layout, a ubiquitous feature of modern digital interaction, holds a fascinating and somewhat counterintuitive history. Originating in the late 19th century for mechanical typewriters, its seemingly haphazard arrangement of keys was intentionally designed not for optimal efficiency, but rather to prevent typewriter keys from jamming by physically separating commonly used letter pairs [42]. Despite its origins in a now-obsolete technology, the QWERTY layout has demonstrated remarkable staying power. It has seamlessly transitioned from typewriters to computer keyboards and, most notably, to the touchscreen interfaces of smartphones. While alternative keyboard layouts have been proposed and tested, the QWERTY layout remains a cornerstone of smartphone interaction [43]. It is not only the most fundamental text entry tool on most smartphones globally [43] but also the most popular choice amongst users [44,45]. As smartphone keyboards—also called soft-keyboards—are a graphical keyboard displayed on a touchscreen [46], this preference stems largely from user familiarity, as the QWERTY layout bridges the gap between personal computer experience and mobile texting habits [45,47].

However, the adaptation of the QWERTY keyboard to the small touchscreen format of smartphones presents inherent user-experience challenges. The limited screen area necessitates miniaturized keys, potentially degrading typing speed and accuracy [48,49,50,51,52]. Furthermore, the wide distribution of keys across the touchscreen means that thumb movements and travel distances vary significantly depending on key position [47,48,52], potentially leading to user discomfort and dissatisfaction [45]. Kang et al. [53] investigated touch performance and user satisfaction with a QWERTY soft keyboard on smartphones, focusing on how key location affects two-thumb typing. The study recruited 33 college students and measured task completion time, touch error frequency, and user satisfaction across 15 keyboard zones. Results showed that zones in the periphery of the keyboard had significantly longer task completion times, while zones in the center were associated with higher user satisfaction. The study suggests that key locations that are easily visible without thumb movement and require less thumb flexion lead to better performance and satisfaction. While touch error frequency did not show significant zonal variation, the study highlights the importance of key placement on QWERTY smartphone keyboards for optimizing user experience.

### 3.2. Types of Mobile Text Entry: Tap vs. Swipe and Intelligent Text Entry (ITE)

The field of text entry on smartphones has been a subject of intense research for decades, driven by the constant need to improve user experience and efficiency [54]. Evaluating the performance and user preference between different technologies is a complex undertaking. Factors such as user skill, cognitive load, the sophistication of autocorrection algorithms, and the vastness of keyboard vocabularies all significantly impact the real-world effectiveness of these methods.

Two dominant approaches have emerged: traditional tap-typing and gesture-based swipe typing. The most familiar method of tap-typing on a QWERTY layout allows users to tap individual keys with a finger or two thumbs. In contrast, Zhai & Kristensson [55] introduced swipe, or gesture typing, which offers an alternative where users glide their finger across the keyboard, tracing a path through the letters of a word. This continuous gesture is then interpreted by the system to predict the intended word, as seen in popular implementations like Swype and Google Keyboard. This approach offers several advantages, including improved typing speed due to the reduced need to precisely target individual small keys on a touchscreen keyboard [56], as well as a relatively low learning curve that makes it easy for users [56,57,58]. Reyal et al. [58] investigated smartphone text entry through a controlled lab experiment and a study in real-world settings. The lab experiment revealed the traditional method to be faster but both methods improved with practice, while the swipe method exhibited higher error rates. Conversely, in the real-world study, with participants using their own devices in their daily lives, the swipe method was demonstrated to be significantly faster, showing greater improvement over time, though still maintaining higher error rates. Notably, the in-the-wild study also revealed a significant trend of users shifting towards the swipe method after experiencing both, suggesting a longer-term preference for the swipe method’s novel interaction style despite its error challenges. To further understand the nuances of swipe typing, Leiva et al. [59] introduced a large-scale dataset specifically designed for shape-writing research. This dataset, encompassing over 11,000 swiped words from over 1300 users, offers a valuable resource for the field and their analysis revealed key insights into swipe typing performance. They found that thumb swiping was surprisingly faster than index finger swiping, users swiped faster on larger screens, and, counterintuitively, increased user experience with swipe typing correlated with higher word error rates.

Beyond specific entry methods, mobile typing presents a distinct set of performance challenges compared to traditional physical keyboards, resulting in generally slower typing speeds [60,61,62]. Research has identified a range of contributing factors that explain this disparity. One primary issue is the virtual nature of mobile keyboards, which lack the tactile feedback of physical keys [49,60,63], hindering touch recognition and increasing reliance on visual attention for accurate input [64]. The small screen size of mobile devices further exacerbates this challenge by necessitating smaller keys, reducing target size, and increasing the precision demands on finger movements [65,66,67] and greater thumbs muscles effort [65]. Compounding these ergonomic limitations is the prevalent use of fewer fingers, often limited to one or two thumbs or a single index finger, in contrast to the multi-finger approach employed on desktop keyboards. Studies consistently demonstrate the speed advantage of two-thumb typing over single-finger input, highlighting the efficiency gained from alternating hand movements and minimizing inter-key intervals [38,43,58,61]. Beyond these device-centric limitations, individual user characteristics significantly shape mobile typing performance. Factors such as age, language proficiency, and prior typing experience create a wide spectrum of typing abilities observed in real-world usage [51,61,63,65,67].

The cultural significance of mobile typing, particularly thumb-typing, has also been analyzed. Ramati [68] provides a compelling analysis of how this technique has emerged as fundamentally reshaped our relationship with text creation. Ramati argues that the shift towards thumb-typing on mobile devices represents a significant departure from the historical emphasis on formal touch-typing developed for typewriters and early computers. Traditional touch-typing focused on finger specialization, accuracy, and efficiency. In contrast, thumb-typing, born from the constraints of mobile interfaces and driven by a culture of instant communication, embraces imprecision and speed. This shift is not merely a technological evolution, but a cultural transformation in how we interact with writing, moving from a paradigm of carefully crafted, edited text towards a more fluid, conversational, and visually enriched mode of communication often characterized by emojis, abbreviations, and a greater tolerance for errors. Furthermore, thumb-typing highlights a shift in the thumb’s functionality, transforming it into an active, executing digit rather than a primarily supporting one, likely influencing the typer’s sensorimotor system.

To mitigate the inherent challenges of mobile typing, Intelligent Text Entry (ITE) methods have emerged as crucial tools to enhance both accuracy and speed [69]. These methods, built upon sophisticated Language Models (LMs), aim to assist users by providing various forms of intelligent assistance. LMs, ranging from traditional N-gram models to complex deep neural networks, analyze language structure to predict the most likely words in a given context. This predictive power enables ITE techniques to perform automatic error correction [70], suggest word completions, and offer alternative typing methods, fundamentally altering the user’s interaction with the mobile keyboard. Word prediction presents a potential avenue for efficiency gains by allowing users to select from a list of suggestions, rather than typing out entire words [69], saving up to 45% of input actions [71]. However, the efficacy of word prediction remains a complex and debated topic, with studies showing mixed results [61,72,73]. While it can reduce keystrokes, the need to shift attention from the keyboard to the suggestion list and the time spent evaluating options can sometimes negate the speed benefits [61,69,72,73]. Palin et al. [61] shed light on the complex and sometimes counterintuitive effects of ITE methods. In their large-scale app-based transcription study, while autocorrection was positively correlated with typing speed, suggesting a performance benefit, word prediction, surprisingly, showed a negative correlation, implying a potential detriment to typing speed.

## 4. Methodological Approaches for Studying Mobile Typing

The rise of digital composition has fundamentally transformed writing research, necessitating a shift in investigative methodologies. While traditional methods focused on the written product, the advent of digital writing and the dominance of keyboarding demand process-oriented approaches to understand the complexities of text production. Accurately assessing typing skills is a significant challenge within writing research. While typing has become the dominant mode of text production in both personal and professional contexts [74], evaluating typing proficiency beyond basic metrics remains complex. The importance of fluent typing in overall writing performance is well-documented [75,76], as researchers emphasize that efficient typing frees up cognitive resources for higher-level writing processes [77].

However, traditional typing tests often rely on limited measures like words per minute and accuracy percentage, which fail to capture the layered nature of typing competence [78,79]. The “layered nature of typing competence” refers to the multiple, interdependent dimensions (e.g., motor fluency, cognitive processing, error correction strategies) that constitute a typist’s overall ability. This underscores the need for more sophisticated approaches that encompass dimensions like accuracy, learnability, and efficiency. Addressing this gap, Wobbrock [80], building upon decades of research in Human–Computer Interaction, systematically categorizes and explains various metrics, offering a valuable resource for researchers and developers in the field. Wobbrock specifically advocates for the unconstrained text entry approach. This methodology mirrors natural text entry by allowing freeform input and backspace-based error correction [81,82,83]. Within this paradigm, numerous measures can be effectively applied to characterize performance.

### 4.1. Key Performance Metrics

For overall method evaluation, aggregate measures are particularly useful (see Table 1). These include Words Per Minute (WPM), a long-established metric [84]. WPM considers the length of the resulting transcribed string and the time taken to produce it, providing a baseline speed measurement. However, its simplicity overlooks the complexities of the entry process. To address this, Adjusted Words Per Minute (AdjWPM) incorporates error penalties, reflecting the trade-off between speed and accuracy [85]. Further enriching the speed dimension, Keystrokes Per Second (KSPS) is a measure of data transfer rate, capturing the user’s action rate regardless of textual output, and Gestures Per Second (GPS) provides a comparable metric for gesture-based methods [80,85]. Analyzing Learning Curves, by modeling WPM over time, can also reveal the learnability of a method [86].

Beyond speed, accuracy assessment involves various error rate calculations. Keystrokes per Character (KSPC) evaluates accuracy during text entry by comparing input keystrokes to the final number of characters in the transcribed string [81]. Minimum String Distance (MSD) quantifies accuracy after entry by measuring the edit distance between the intended and produced text [81,87,88]. For a more granular error analysis, Corrected, Uncorrected, and Total Error Rates [83] categorize errors based on correction status, offering a unified error metric. In scenarios with clustered errors, Cumulative and Chunk Error Rates [85] provide alternative counting methods.

Efficiency measures further refine the evaluation. Metrics such as the KSPC characteristic measure [89,90] offer a model-based quantification of inherent method efficiency by calculating theoretical keystrokes per character. Other related measures focus on the error correction process: Correction Efficiency assesses the effectiveness of correction mechanisms, and Participant Conscientiousness reflects user diligence in error correction [83]. Additionally, Utilized and Wasted Bandwidth [83] quantify the proportion of keystrokes contributing to correct text, while the Cost per Correction (CPC) [91] evaluates the associated effort.

Character-level measures provide even finer-grained insights. Intra- and Intercharacter Time offer character-level analogs to entry rate, revealing typing rhythm and pauses [30]. Analyzing Uncorrected Errors at the character level allows for the identification of specific error types like substitutions, insertions, and omissions [82,90]. Furthermore, examining Corrected Errors in Input Streams captures the nuances of user error correction behavior, differentiating between “corrected-and-wrong” and “corrected-but-right errors” [82].

To meticulously study writing dynamics, keystroke logging is a pivotal technique. It records every keystroke, mouse click, and related action during text production, providing a time-coded stream of data for precise reconstruction of the writing process [92]. This unobtrusive method offers valuable insights into the cognitive and motor processes, particularly in controlled experiments.

**Table 1:** Metrics capturing different dimensions of performance to evaluate typing skills.

Category	Metric Name (Acronym)	Description	Key Source(s)
Overall/Speed	Words Per Minute (WPM)	Baseline speed measurement based on output text.	Yamada (1980) [84]
	Adjusted Words Per Minute (AdjWPM)	Incorporates error penalties to reflect the trade-off between speed and accuracy.	Matias et al. (1996) [85]
	Keystrokes Per Second (KSPS)	Measure of data transfer rate, capturing user’s action rate regardless of textual output.	Wobbrock (2007) [80]
	Gestures Per Second (GPS)	Comparable metric to KSPS specifically for gesture-based methods.	Wobbrock (2007) [80]
	Learning Curves	Modelling metrics over time to reveal the learnability of a method.	Card et al. (1983) [86]
Accuracy	Keystrokes per Character (KSPC)	Evaluates accuracy by comparing input keystrokes to transcribed characters.	Soukoreff & Mackenzie (2001) [83]; MacKenzie (2002) [89]
	Minimum String Distance (MSD)	Quantifies accuracy after entry by measuring the edit distance between intended and produced text.	Soukoreff & Mackenzie (2001) [83]; Wagner & Fischer (1974) [87]
	Corrected Error Rate	Proportion of errors corrected by the user.	Soukoreff & MacKenzie (2003) [83]
	Uncorrected Error Rate	Proportion of errors remaining in the final output text.	Soukoreff & MacKenzie (2003) [83]
	Total Error Rate	All errors made, regardless of correction status.	Soukoreff & MacKenzie (2003) [83]
Efficiency	Correction Efficiency	Assesses the effectiveness of correction mechanisms.	Soukoreff & MacKenzie (2003) [83]
	Participant Conscientiousness	Reflects user diligence in error correction.	Soukoreff & MacKenzie (2003) [83]
	Utilized Bandwidth	Proportion of keystrokes contributing to correct text.	Soukoreff & MacKenzie (2003) [83]
	Wasted Bandwidth	Proportion of keystrokes that do not contribute to correct text (e.g., errors, corrections).	Soukoreff & MacKenzie (2003) [83]
	Cost per Correction (CPC)	Evaluates the effort associated with the error correction process.	Gong & Tarasewich (2006) [91]
Temporal/Character- Level	Intra-character Time	Time elapsed *within* the production of a single character.	Rumelhart & Norman (1982) [30]
	Intercharacter Time	Time elapsed between successive keystrokes; reflects typing rhythm and pauses.	Rumelhart & Norman (1982) [30]; Wengelin (2006) [93]
	Uncorrected Errors at character level	Identification of specific error types (substitutions, insertions, omissions) at the character level	MacKenzie & Soukoreff (2002) [90]; Wobbrock & Myers (2006) [82]
	Corrected Errors in Input Streams	Captures nuances of correction behavior (e.g., “corrected-and-wrong”, “corrected-but-right”).	Wobbrock & Myers (2006) [82]

Within keystroke logging data, the parameter interkey intervals (IKIs), also known as ‘interkey latencies,’ emerges as a particularly insightful metric. IKIs represent the time elapsed between successive keystrokes and are crucial cognitive indicators in writing research [93]. Shorter IKIs generally reflect fluent, automatized typing, while longer IKIs, or pauses, can signify cognitive processing, planning, or revisions during text composition. The analysis of IKI distributions and variations therefore offers a window into the cognitive demands of writing, revealing how writers manage the complex interplay of motor skills, linguistic encoding, and higher-level cognitive processes [94]. However, interpreting these pause patterns is not straightforward due to the inherent variability in typing proficiency across individuals. Therefore, accurately assessing typing skills becomes paramount for researchers aiming to dissect the cognitive underpinnings of writing and to develop more nuanced and informative measures beyond basic metrics like speed and accuracy [78,79]. The challenge lies in developing instruments that can effectively capture and account for this inherent variability in typing competence to provide a complete and more accurate picture of the writing process.

### 4.2. Controlled Laboratory Tasks

The most common procedure for assessing typing skills, especially in controlled settings, is based on transcription tasks, particularly copy-typing tasks. In this type of study, participants are shown predetermined phrases one at a time and asked to transcribe them as quickly and accurately as possible [90,95]. These phrases are usually selected randomly from a predefined set, with the most commonly used being the Enron Mobile Email Database [96,97] and the MacKenzie Database [98], which contain memorable everyday sentences typically typed on mobile devices. This method helps avoid common issues associated with free text entry in user studies, which can compromise experimental control and accuracy assessment due to the absence of a reference text and a lack of control over performance measurements. In contrast, using predefined phrases, as in copy-typing tasks, ensures reliable performance evaluation and reproducibility [95]. Such studies can be conducted entirely in a laboratory, in a controlled setting and using devices provided by researchers for accurate quantification of typing speed and errors [83].

Azenkot and Zhai [43] conducted a copy-based transcription task and found that text entry speed varied notably depending on the input method: participants reached approximately 50 WPM using two thumbs, roughly 36.3 WPM with one thumb and around 33.8 WPM when typing with the index finger. Notably, error rate was higher while typing with two thumbs. More recently, Van Waes et al. [99] focused on the creation and validation of a standardized laboratory-based copy-typing task, utilizing a substantial corpus of copy tasks, in order to develop a reliable and sensitive tool capable of capturing individual typing skills across varying textual conditions. This methodological investigation involved analyzing a large-scale dataset of keystroke data to explore the influence of factors like age and task characteristics on typing performance, with a particular focus on inter-key intervals (IKIs). Notably, the study found that copying speed exhibited a non-linear relationship with age, peaking in the 21–30 age range before a gradual decline. Furthermore, the analysis of inter-keystroke intervals across different copy-task components (Tapping, Sentence, Word combinations, Consonants) revealed distinct performance patterns, demonstrating sensitivity to task demands. Crucially, the study established the test–retest reliability of the copy task, confirming its consistency as a measurement tool. The application of Bayesian mixture models effectively captured the mixture of fluent and disfluent keystroke transitions inherent in typing data. This allowed for a more nuanced understanding of typing, revealing that fluent copy-typing was less influenced by lexical information than bigram frequency, while disfluent keystrokes were more strongly associated with a lack of lexical context.

Beyond transcription, composition tasks offer greater ecological validity by mirroring how real-world users predominantly compose original content rather than transcribing existing text [100]. To mitigate this, researchers have adopted composition tasks to evaluate text entry performance [100,101,102], proposing task frameworks designed to replicate more authentic typing behaviors in controlled environments. Examples include responding to predefined mobile notifications, generating messages under simulated mobile contexts, composing freeform messages, or emulating interactions via assistive technologies [100]. Nicol et al. [103] extended this approach with an image-description task. While providing instructions to prompt spontaneous text generation [100,102] is successful, composition tasks introduce methodological challenges. Significant variability across participants and elevated cognitive demands may undermine reliability and internal validity [100]. Performance metrics are skewed by time dedicated to content planning rather than input interaction, while the absence of reference texts prevents systematic error quantification. Moreover, limited participant creativity frequently produces rudimentary or abbreviated outputs, diminishing ecological validity. These constraints collectively weaken experimental precision, indicating that transcription tasks or standardized protocols might more effectively isolate text entry performance during evaluation.

### 4.3. Real-World Data Collection

While controlled laboratory settings offer precision, real-world data collection methods provide ecological validity. One such approach, extensively used in text entry studies, is the experience sampling method. Here, participants are prompted throughout the day to complete text-entry tasks, usually via web or by a customized application, using their own smartphones in real-world contexts. This approach allows for more ecologically valid data, as it better reflects participants’ typical typing performance outside of a laboratory environment. Moreover, the online nature of this type of experiment, combined with the possibility of involving unsupervised participants, allows researchers to conduct large scale studies [61,104].

A different approach allowed participants to complete an online transcription task at their convenience [61]. The study involved over 37,000 volunteers, assessing metrics such as words per minute, uncorrected error rate, and keystrokes per character. Results showed an average typing speed of 36.2 WPM. Notably, typing speed was 15 WPM slower compared to the typing on a physical keyboard examined using the same methodology by Dhakal et al. [36]. Additionally, a comparison between the two typing modes revealed that participants were more likely to leave errors uncorrected when using mobile keyboards. This is likely due to the higher effort required to correct mistakes on mobile devices and the more constrained text editing options available [61]. Alongside performance data, Palin’s study [61] collected rich demographic and behavioral information through a questionnaire, encompassing age, gender, language proficiency, typing habits, and ITE usage. Their findings revealed a negative correlation between age and typing speed and that two-finger typing (typically thumbs) was significantly faster than one-finger typing. Other studies have adopted similar real-world data collection methods by integrating transcription tasks into serious mobile games [105,106,107].

A comparative study by Reyal et al. [58] examined text entry performance across laboratory and an experience sampling experiment. They concluded that both methods offer unique insights and are most valuable when used together for a more comprehensive understanding. However, traditional copy tasks presented without context can be tedious, provoking mental fatigue and leading to reduced engagement [108,109]. To address this, Komninos et al. [110] proposed an online transcription task where phrases were presented within conversational contexts. They investigated how the transcription performance might be affected by the presentation of high and low memorability phrases when shown either alone or accompanied with conversational prompts (Large Language Model generated or written by human researchers). Results showed that while prompts did not influence input speed, they significantly improved recall for low-memorability phrases, resulting in fewer uncorrected errors. Participants also reported greater task engagement, perceived realism, and emotional involvement.

Another robust real-world data collection methodology is the passive sensing approach. Unlike experience sampling, where participants are prompted, passive sensing involves installing background services or system-wide tools (e.g., customized keyboards) on users’ devices to collect natural typing data from everyday tasks without actively prompting the user to perform specific typing tasks. This method offers a powerful way to observe typing behavior in highly ecological settings, capturing data from various situations—indoors or outdoors, quiet or noisy environments, stationary or in motion. All these contextual factors can alter user performance and are referred to as situationally induced impairments and disabilities [111]. Akpinar et al. [112] identified five such contextual dimensions (environment, mobility, social context, multitasking, and distraction), noting limited research on their effects on smartphone typing performance. Passive sensing helps overcome the limitations of laboratory-based and traditional experience sampling studies in this regard [58,113,114,115,116].

Buschek et al. [117] presented an Android keyboard application and data logging concept designed to study free-typing behavior in real-world contexts, including the influence of smart features like autocorrection and word suggestions. Their three-week field study with 30 participants analyzed 5.9 million keystrokes, revealing users achieved an average typing speed of 32.1 WPM in everyday use. Furthermore, their analysis showed significant variations in typing behavior across different app categories, with messaging apps exhibiting the highest usage of word suggestions. Komninos et al. [113] investigated how mobile input errors emerge in real-world situations and how users respond, finding that although participants slowed down and used spellcheckers, spelling errors remained common, and finger slippage was not a major error source. Nicolau et al. [114] found that blind users employing touchscreen screen readers were faster and committed more errors during everyday typing compared to laboratory typing sessions. Similar results were found in a passive sensing study on desktop computers [118].

Rodrigues et al. [115] conducted a month-long user study comparing three data collection methods: transcription-based experience sampling, composition-based experience sampling and passive sensing. Results showed significant differences in typing performance, behavior metrics, and user experience across methods. Participants typed fastest during the transcription task (53.3 WPM observed online, 47.8 WPM without observation), slowest during the composition task (36.9 WPM), and at an intermediate speed with passive sensing (41.5 WPM). Error rates were higher during composition and passive sensing. Furthermore, users tended to use more suggestions and autocorrections during passive sensing. However, the slower input speed during passive sensing, compared to transcription, contrasts with precedent research [114,118], although those studies also found higher error rates during passive sensing. Taken together, these results indicate that typing performance and behavior differ notably across data collection methods [115].

Despite their strengths, these data collection methods each have limitations. Controlled laboratory settings, while valuable for rigorous investigation, may not fully capture the complexity, variability, and ecological validity of everyday mobile typing behavior. The artificially constrained lab environment starkly contrasts with the dynamic and often distracting contexts of real-world text entry. Furthermore, using researcher-provided devices often fails to reflect personalized typing experiences. Transcription tasks, commonly employed for experimental control, also fall short of representing the complexities of free text composition, where planning, revision, and communication goals significantly shape typing behavior. Finally, the deliberate exclusion or simplification of intelligent text entry methods in lab settings overlooks the critical role and adaptive influence of features like autocorrection and word suggestions in contemporary mobile typing. Conversely, real-world data collection, particularly passive sensing from users’ personal smartphones, introduces a complex array of technical and ethical challenge [117]. Technical limitations range from inconsistent keycode reporting to inability to reliably capture multi-touch events. Privacy concerns also represent a paramount challenge [117], as typing data is inherently personal. Therefore, combining approaches often yields the most comprehensive understanding.

To provide a comprehensive understanding of the diverse research landscape in mobile typing, Table 2 offers an overview of the various data collection paradigms employed. This structured summary highlights the key characteristics and primary contributions of each method, from controlled laboratory tasks to naturalistic real-world data collection.

## 5. Mobile Typing as a Behavioral Biomarker

### 5.1. Smartphone Interaction Dynamics: Applications in Aging, Affective States, and Neurological Health

Beyond assessing skill and performance, smartphone interaction patterns are increasingly recognized for their potential as behavioral biomarkers for health monitoring. Very recently, a new metric has been identified. Ceolini et al. [119] conducted a pioneering study investigating the potential of smartphone interaction patterns as a biomarker for healthy and pathological aging. Central to their innovative approach was the use of “behavioral age”, a novel dependent variable derived from smartphone touchscreen interaction dynamics. This “behavioral age” served as a quantifiable marker of an individual’s aging status, inferred from the complex patterns of their daily digital behavior on their smartphones, rather than chronological years. To capture these daily digital behaviors, the researchers utilized a background application, TapCounter (QuantActions, Lausanne, Switzerland) [120], installed on Android operating smartphones. This app meticulously recorded the timestamps of all touchscreen interactions with millisecond resolution, ensuring a granular capture of user behavior.

To create the “behavioral age” metric, researchers developed a “Behavioral Age Model.” This model employed a scalable machine learning system utilizing decision tree regression, trained on a large-scale longitudinal dataset. It leveraged several independent variables to estimate chronological age, including self-reported gender, screen size, the number of smartphone interactions, entropy, and the joint interval distribution (JID) of touchscreen interactions. The JID, a crucial input, was estimated by accumulating inter-touch intervals (ITI) and then applying 2D kernel density estimation to the log10-transformed space of each ITI and its subsequent interval. This distribution, capturing the nuanced dynamics of next-interval interactions, was foundational for calculating behavioral age. The model, validated through rigorous cross-validation, achieved remarkable accuracy in age prediction, demonstrating the potential of smartphone interaction patterns to serve as a proxy for age-related changes.

Specifically, extending their analysis to individuals with stroke and epilepsy, the researchers applied this healthy aging model to these neurologically distinct populations. A significant finding was that both stroke survivors and people with epilepsy [121] exhibited an “advanced behavioral age” compared to their healthy, age-matched counterparts. Subsequently, by applying this model daily to individual user data, Ceolini et al. [122] were able to track fluctuations in behavioral age over time, enabling them to investigate the dynamic nature of aging and the concept of behavioral resilience within the context of everyday digital life, with behavioral age serving as their central dependent variable.

Further possibilities emerge from data collection obtained by smartphone passive sensing background applications. In a study by Wampfler et al. [123], it was demonstrated the feasibility of accurately predicting user affective states during real-world smartphone usage through passively collected data. The study employed a large-scale, in-the-wild data collection approach, recruiting 82 participants over 10 weeks to use a custom Android application. This application passively recorded a rich dataset encompassing keystroke-level touch data, inertial sensor data (accelerometer, gyroscope, magnetometer, proximity, light, step counter), device usage logs, and user context. Participants provided self-reports of their affective states (valence, arousal, dominance, basic emotions, and stress) using a user-friendly emoji-based interface integrated into the custom keyboard. A combination of time-based and event-based triggers prompted self-reports throughout the day to capture natural fluctuations in effect. This rich dataset, encompassing keystroke dynamics, inertial sensor readings, and user context, was processed using an innovative feature engineering technique based on two-dimensional heat maps, which effectively encoded complex behavioral patterns. These heat maps served as input to a deep convolutional neural network model trained to classify affective states. The resulting model achieved remarkable performance, accurately predicting valence and arousal, and demonstrating that sensor data alone could provide comparable results to keystroke data, offering a more privacy-conscious alternative.

Similar studies involving typing tasks and keystroke dynamics analysis have shown strong potential in health research [124]. These measures have emerged as promising biomarkers for the early identification and monitoring of impairments associated with neurological disorders such as Parkinson’s disease [125,126,127] and multiple sclerosis [128,129,130]. Furthermore, they have been shown to effectively capture depressive symptoms [131,132], affect in suicidal ideation [133], mood variability in bipolar disorder [134,135], mental fatigue [136] as well as in providing information on the cognitive functioning of individuals with mood disorders [137]. Along these lines, Akeret et al. [138] propose smartphone-based behavioral analysis as an innovative, objective, and inexpensive alternative for continuous monitoring. Given the widespread use of smartphones, they argue that passively collected data, such as touchscreen interactions and usage patterns, can reflect an individual’s functional level in real-time.

### 5.2. Neurophysiological Evidence for Sensorimotor Plasticity

Building upon this behavioral perspective, recent neurophysiological studies have begun to explore the direct impact of smartphone interaction on brain function and cortical organization. Gindrat et al. [8] utilized electroencephalography (EEG) and objective battery log data to investigate use-dependent cortical plasticity in touchscreen phone users. Comparing touchscreen users to non-users, they found that touchscreen users exhibited enhanced cortical potentials in response to tactile stimulation of their fingertips, particularly the thumb and index finger, with the magnitude of these potentials correlating with the intensity of phone use. This highlighted that the repetitive movements inherent in touchscreen interaction reshape sensory processing in the hand, specifically within the primary somatosensory cortex (S1), suggesting a remarkable adaptation of the sensorimotor cortex to daily digital technology use.

Furthermore, extending this line of inquiry beyond broad use-dependent plasticity to the nuances of action itself, Kock et al. [139] investigated the neural processing of different types of movements during natural smartphone interaction. By combining EEG, movement sensors, and artificial neural networks to classify goal-directed (touchscreen interaction resulting) and non-goal-directed thumb movements, they revealed distinct neural signatures for these seemingly similar actions. Goal-directed movements were characterized by robust motor preparation and post-movement processing, which in EEG signals manifested as contralateral sensorimotor activity (including slow rising positivity and subsequent negativity), suggesting the involvement of primary motor cortex (M1), premotor cortex (PMC), and supplementary motor area (SMA) for planning and executing complex motor sequences. In contrast, non-goal-directed movements showed dampened cortical engagement, with attenuated post-movement negativity and only marginal beta-band desynchronization over sensorimotor areas. Critically, these non-goal-directed movements uniquely evoked error-related neural signals, such as frontal negativity mimicking the error-related negativity (ERN), strongly indicating the involvement of prefrontal cortical areas (PFC) in monitoring and processing unexpected outcomes.

Beyond the direct EEG evidence presented by Kock et al., the very nature of mobile typing, involving rapid and precise finger movements on a virtual interface, inherently implicates other key brain regions. The posterior parietal cortex (PPC) is crucial for visuospatial processing and eye–hand coordination, essential for navigating the virtual keyboard. The cerebellum contributes significantly to fine motor coordination, timing, and error learning, while the basal ganglia are fundamental for the automatization of motor skills and procedural learning, which are vital for typing fluency. Kock et al. themselves allude to the role of subcortical structures in preparing parallel actions [139], further supporting the inclusion of these regions. Given the inherently multimodal nature of mobile typing, which relies heavily on visual input from the keyboard and typed text (engaging occipital areas) and linguistic processing for word recognition and comprehension (involving temporal areas), it is challenging to definitively exclude any brain region a priori from its complex neural network. This highlights the integrated nature of sensorimotor and cognitive functions underlying digital interactions, encompassing perception, motor planning and execution, and higher-level cognition.

While EEG provides excellent temporal resolution for studying dynamic neural processes, future research could greatly benefit from incorporating other neuroimaging modalities. Functional Magnetic Resonance Imaging (fMRI) offers superior spatial resolution, allowing for precise localization of cortical and subcortical regions involved in mobile typing, including those implicated in motor sequence learning. Moreover, Functional Near-Infrared Spectroscopy (fNIRS) presents a portable alternative to fMRI, making it particularly suitable for investigating brain activity during mobile typing in ecologically valid, real-world settings, despite its lower spatial resolution. Furthermore, given the significant transformation of the thumb’s role from a supporting digit to an active effector in complex motor sequences (as highlighted in our Introduction), techniques like Transcranial Magnetic Stimulation (TMS) are indispensable. TMS can be used to non-invasively probe the excitability and reorganization of the primary motor cortex (M1) representation of the thumb, offering direct evidence of cortical plasticity and functional changes related to its intensive use in mobile typing. The integration of these techniques could provide a more comprehensive understanding of the neural networks and their plasticity associated with digital interactions, enhancing the utility of mobile typing as a behavioral biomarker for various conditions.

These findings underscore that even within the seemingly mundane activity of smartphone use, the brain subtly but distinctly differentiates between purposeful and inconsequential actions, indicating a sophisticated level of neural processing underlying our daily digital behaviors and demonstrating the power of combining smartphone-derived behavioral metrics with neurophysiological measures to gain a deeper understanding.

## 6. Conclusions

Mobile typing has emerged as a ubiquitous, complex, and ecologically relevant motor activity in everyday life; nevertheless, it is only recently being systematically explored as an object of scientific inquiry. Mobile typing, although seemingly a simple action, represents an integrated behavior in which sensorimotor, cognitive, and affective processes converge, making it a privileged target for simultaneously examining multiple functional levels of human behavior.

This narrative review highlights significant progress in understanding mobile typing, yet several pressing theoretical, methodological, and empirical research gaps persist, requiring dedicated future attention.

Theoretically, a central issue concerns the origin and nature of mobile typing ability: whether it primarily derives from the generalization of previously acquired motor patterns or involves significant de novo learning. De novo learning, in the context of human-technology interaction, refers not necessarily to the acquisition of entirely novel kinematic patterns, but rather to the learning of new functional meanings or goal-action mappings for existing physical movements within a digital environment. This contrasts with motor adaptation, where existing motor skills are modified to suit new environmental demands while maintaining a similar functional outcome. Indeed, some evidence suggests that certain motor programs, such as bimanual alternation, motor chunking, and bigram sensitivity are preserved across different interfaces [38], aligning with the view that the motor system selects and generalizes action patterns based on goals [140]. However, recent studies documenting heterogeneity in individual motor strategies and learning trajectories on mobile devices [37,39,40,59] underscore an open question: what individual and contextual factors guide the development and deployment of these motor programs. This theoretical challenge is particularly pertinent when considering distinct developmental trajectories across generations, as highlighted in the Introduction. The established divergence in sensorimotor repertoires, formed either predominantly by physical interaction (older adults) or through concurrent physical and digital engagement (younger generations), raises crucial questions about the nature of sensorimotor learning in the digital age. Here, ‘physical interaction’ refers broadly to all naturalistic engagement with the non-digital environment, from manipulating objects to complex skilled movements. For example, for Digital Immigrants, a movement like a horizontal thumb swipe might traditionally have been associated with actions such as removing lint or a crumb from a surface in the physical world. When performing a similar kinematic motion on a smartphone for an entirely new digital purpose—such as swiping to select an accent mark, browsing photos, or navigating through TikTok videos—the key question arises: is this an adaptation of the existing movement to a new context, or does it require de novo learning of the functional link between that specific physical action and its distinct digital outcome? Conversely, for Digital Natives, these goal-action mappings might be integrated as primary motor skills from an early age, forming part of their intrinsic sensorimotor repertoire. Understanding the neural mechanisms underlying the acquisition of these new skill representations and functional re-mappings is crucial for the advancement of motor learning research. The extent to which these fundamentally different formative experiences lead to distinct sensorimotor and cognitive architectures, and how they impact digital interaction, remains a critical, underexplored area.

Methodologically, while existing research has provided insights into aspects such as finger trajectories and eye-hand coordination, the field currently lacks the necessary fine-grained analysis of single-typing action, the goal-directed movement of a finger from an initial key position to depress the intended target key, to fully understand the motor control of individual finger movements at the micro-level. This is particularly crucial for the thumb, given its critical function in mobile text entry. For many, especially younger users, mobile typing has transformed the thumb from primarily an oppositional digit to an active effector in complex motor sequences. This shift in functional role likely entails significant reorganization of its cortical representation, warranting in-depth investigation. To effectively address this, and more broadly to capture the nuances of individual thumb movements, studies employing high-precision tools like 3D motion capture to quantify the precise dynamics of thumb strokes are critically needed. Such granular data, including detailed metrics like peak velocity, deceleration patterns, and exact spatial and temporal profiles of thumb movements, are vital for building accurate and predictive models of motor planning and execution [14,15], offering new insights beyond the more general parameters studied so far (e.g., inter-key intervals). Furthermore, the current heterogeneity of metrics across mobile-specific studies—though reflective of diverse research questions—hinders cross-study comparability and cumulative knowledge building. The identification of a shared core set of indicators, alongside common protocols for data collection and analysis, is a necessary prerequisite for the theoretical and applied advancement of this domain [80,83].

Empirically, beyond motor program analysis, mobile typing provides access to cognitive-level metrics. High-resolution temporal parameters—such as inter-key interval—enable researchers to capture not only speed and accuracy but also markers reflecting planning-related demands and motor control structure [99]. Moreover, these metrics have shown sensitivity to situational variables such as affective state [123], as well as to stable individual differences like age and cognitive profile [119], thus supporting the use of typing as a digital biomarker for the low-burden assessment of motor and cognitive performance. These behavioral findings are also supported by neurophysiological evidence. Repeated mobile use has been associated with use-dependent plasticity in the somatosensory representation of the fingers [8], while EEG studies have shown that the brain can clearly differentiate between intentional and unintentional touches, even when the kinematic features are superficially similar [139]. These results underscore the relevance of mobile typing to investigate the dynamic interaction between automatic processes, intentional actions, and voluntary motor regulation. Crucially, mobile typing offers a unique and highly monitorable model for investigating sensorimotor skill learning, development, and potential decline due to age or pathology, providing a refined and easily measurable instrument for continuous assessment in ecological settings. Indeed, changes in typing patterns are emerging as early indicators of neurological vulnerability, suggesting potential clinical applications in diagnosis and monitoring [130,131]. Therefore, longitudinal designs could elucidate how and when individual strategies emerge, stabilize, and change throughout the lifespan (including developmental stages) or in response to neuropathological conditions. Further empirical work is also needed across diverse populations and under varying ecological conditions to enhance the generalizability and practical applicability of findings.

Ultimately, the practical implications of this research are substantial and far-reaching. A deeper understanding of mobile typing can directly inform the design of more intuitive, adaptive, and inclusive digital interfaces, improving user experience for the general population. It can also lead to the development of more effective assistive technologies for users with motor or cognitive impairments [111,114]. Furthermore, refining the use of mobile typing data as robust behavioral biomarkers holds immense promise for health monitoring and the early diagnosis of neurological and mental health conditions [119,130,131]. The future of this subfield lies in integrating high-resolution behavioral data with neurophysiological measures [139], developing standardized methodologies, and fostering interdisciplinary collaboration to fully capture and understand the dynamic sensorimotor and cognitive processes involved in digital interactions. This approach leverages the ecological nature of mobile typing to contribute to the theoretical understanding of human–technology interaction, which itself can serve as an extremely useful and effective tool for investigating human–world interaction. This is particularly true given that many goals previously achieved through interaction with the physical world (e.g., browsing a photo album, going to the bank to make a transfer) are now accomplished via technology (e.g., scrolling through photos in a phone gallery, making a transfer using a banking app).

In conclusion, mobile typing stands as a compelling model for advancing our understanding of human sensorimotor learning, development and cognitive function, providing a rich, ecologically valid platform for investigating human–world interaction as it is increasingly mediated by digital technologies, with profound implications for science and society.

## Figures and Tables

**Table 2:** Overview of data collection paradigms for studying mobile typing dynamics.

Category of Approach	Methodologies and Key Characteristics
Controlled Laboratory Tasks	Description: Research conducted in a controlled environment, often using researcher-provided devices. Focus: Baseline performance, skill acquisition, controlled variable manipulation. Examples: Copy-typing tasks (transcribing standard phrases like Enron Mobile Email Database [96,97], MacKenzie Database [98]), composition tasks (generating freeform text or responses to prompts). Characteristics: High experimental control, precision, reproducibility. Limitations: Artificial context, limited ecological validity, potential for mental fatigue
Real-World Data Collection	Description: Data collected from users in their natural daily environments. Focus: Typical performance, contextual factors influencing behavior, long-term monitoring. Examples: Experience Sampling Method (ESM) (users prompted for tasks on their own smartphones), Passive Sensing (background apps/custom keyboards unobtrusively collect typing data from everyday use). Characteristics: High ecological validity, allows for large-scale studies, captures naturalistic context (e.g., environment, mobility). Limitations: Lower experimental control, technical/ethical challenges (e.g., privacy, data consistency, noise from real-world contexts)
